# Of Puberty and the Critical Age of Women, and of the Periodical Discharge of Ova by Women and the Mammiferæ

**Published:** 1845-04

**Authors:** 


### BIBLIOGRAPHICAL NOTICES.

"*Positive Theory of the Fecundation of the Mammiferæ.* By F. A. Pouchet, M. D., Paris, 1842."

"*Of Puberty and the Critical Age in Women, and of the Periodical Discharge of Ova by Women and the Mammiferæ.* By M. A. Raciborski, M. D. &c., Paris, 1844, pp. 520."

